# The role of matrix extracellular proteins and metalloproteinases in head and neck carcinomas: an updated review

**DOI:** 10.1016/S1808-8694(15)31289-1

**Published:** 2015-10-20

**Authors:** Antonio L.A. Pereira, Simone S.L. Veras, Éricka J.D. Silveira, Flávio R.G. Seabra, Leão Pereira Pinto, Lélia B. Souza, Roseana A Freitas

**Affiliations:** 1 Professor of Periodontics, Department of Dental Sciences, Federal University of Maranhão; Ph.D. studies in Oral Pathology under course, Federal University of Rio Grande do Norte (UFRN); 2 Professor of Oral Pathology, Department of Pathology and Dental Clinical Practice, Federal University of Piauí, Ph.D. in Oral Pathology under course,/UFRN, Sponsored by CNPq; 3 Ph.D. in Oral Pathology under course, UFRN; 4 Professor of Periodontics and Surgery, Universidade Potiguar, Ph.D. in Oral Pathology under course, UFRN; 5 Faculty Professor, Program of Post-Graduation in Oral Pathology, UFRN; 6 Professor, Ph.D., Post-graduation in Oral Pathology, UFRN

**Keywords:** head and neck carcinomas, matrix extracellular, matrix metalloproteinase

## Abstract

Interactions involving tumor cells and the extracellular matrix (ECM) strongly influence tumor development, including head and neck tumors, affecting cell proliferation and survival as well as the ability to migrate beyond the original location into other tissues to form metastases. These cell migration is often facilitated by partial destruction of the surrounding ECM, which is catalyzed by matrix metalloproteinases (MMPs), a family of more than 20 endopeptidases that is controlled by regulated expression of specific inhibitors (TIMPs). Several studies of ECM and MMPs markers have provided additional diagnostic and prognostic information in head and neck carcinomas. In this review, we are considering the role of ECM and MMPs in tumor progression, emphasizing its proteolytic contributors to this process, and interactions between several members of ECM providing substrate to regulation of this process.

## INTRODUCTION

Squamous cell carcinoma is the most common head and neck malignant neoplasm, which is characterized by presenting aggressive clinical course and significant likelihood of developing metastases, especially for neck lymph nodes^1^.

Carcinogenesis of tumors of the head and neck is a process of multiple steps, in which there are genetic affections that leads to deregulation of cell metabolism. Aggressiveness of tumors is related to many different factors, among which we can refer to level of histological malignancy, side of lesion, degree of adjacent tissue involvement, presence of metastasis at diagnosis and anatomical location of tumor. One of the main factors that influence prognosis of these neoplasms is presence of metastasis of neck lymph nodes or distant organs. The assessment of factors that influence this process is important for better knowing about tumor behavior and development of anti-cancer therapies.

Tumor growth results from imbalance between cell proliferation and apoptosis and it is influenced by angiogenesis, whereas metastatic potential is influenced by affections to cell-cell and cell-matrix interactions.

Extracellular matrix is a structure consisted of many proteins and polysaccharides distributed differently among the many different tissues of the body. This environment provides appropriate conditions for growth and cell differentiation, favoring the survival of tissues.

Basic constitution of matrix is fibrous proteins such as collagen and elastin, and elongated glucoproteins such as fibronectin and laminin, whose function is to provide cell-matrix adhesion, in addition to glucoaminoglican and proteoglican that form a bed of gel, in which there all the constituents of the matrix^2^.

The role of extracellular matrix in the tumor microenvironment is not limited only to acting as a physical barrier to neoplasm, but it also works as a reservoir for ligant proteins and growth factors that influence its behavior^3^.

Degeneration of matrix is a key event in invasion and metastasis of tumors. This degradation is caused by proteolytic enzymes, denominated metalloproteinase, which act by disorganizing the matrix through a process that affects cell-cell and cell-matrix interactions.

The present study aims at revising the role of extracellular matrix in head and neck carcinoma, comprising the mechanisms that take place during its degradation and favoring the process of invasion and metastasis, which are the main factors that influence prognosis and survival of patients with this neoplasm.

## LITERATURE REVIEW

### Extracellular matrix (ECM): Structure and Function

Animal and vegetable tissues are not formed only by cells, but also by extracellular space filled with a complex of fibrous and proteic components named extracellular matrix (ECM)^2^, ^4^. It comprises varied proportions of proteins and polysaccharides, organized in networks, which are responsible for the morphological, functional and pathological diversity of tissues, providing the appropriate substrate for growth and differentiation of varied cell types^4^, ^5^.

Among the constituents of ECM we can include many different types of macromolecules (proteoglicans and glycosaminoglicans) in addition to fibrous proteins of collagen and elastin, both with structural and functional aspects, and as adhesive glucoproteins such as laminin, tenascin and fibronectin^6^.

ECM is classified as interstitial matrix, which comprises the whole space existing between mesenchymal cells and basal membrane, which is represented by specialized extracellular matrix in laminar shape^2^, ^7^. The interaction between the protein and fibrous complex, soluble proteins and surface receptors influence many cell properties, including migration, proliferation, stages of differentiation and apoptosis^4^.

Interactions and links between cells and ECM components are conducted by specific receptors named integrins, which are transmembrane proteins, with extracellular domain that is attached to ECM components and a cytoplasmatic component linked to the cytoskeleton portion formed of actin^8^, ^9^.

### ECM x Cancer

It has been clearly established in the literature that tumor stroma is directly related with biological behavior of neoplasm, despite the fact that for a long time, neoplasm cells were the main focus of cancer studies^10^.

The capability malignant cells have to destroy basal membrane and the other components of ECM has been related to invasive potential of this neoplasm^11^. Recent studies conducted using markers of ECM components such as enzymes related to its degradation (MMP) have contributed to the understanding of the biological and clinical behavior of head and neck carcinoma.

In the studies by Thomas et al.^12^ the increase in production of these enzymes was associated with metastatic phenotype and it was invasive in many tumors, and consequently, with tumor behavior and prognosis.

ECM influences the behavior of neoplasms through many mechanisms related to proliferation, progression and tumor invasion. For example, local invasion and development of metastases in head and neck carcinoma are directly associated with extracellular matrix, including basal membrane affection and induction of production of many ECM proteins^13^.

When head and neck squamous cell carcinoma are in development, adjacent tissue suffers many variations such as stroma lysis with degradation of ECM, a fact considered to be the preparation for tumor invasion^12^, ^14^. Additionally, such structure may have a more active role during carcinogenesis to induce angiogenesis and production of growth factors, stimulating proliferation of neoplastic cells^10^, ^15^.

During the process of tumor invasion, neoplastic cells cross two types of matrix (basal membrane and interstitial stroma) and biochemical reactions between normal cells and ECM influence the process of tumor invasion in neoplasm^7^, ^16^.

According to Sherstha et al.^17^ ECM constituents contribute directly or indirectly to the tumor process, owing to the fact that the structure has potentially anti-adhesive components, modulators of adhesion, proliferation and cell migration. Additionally, the authors reported that ECM may regulate cell behavior using different mechanisms: first through composition of proteins in a specific tissue and second in synergic interactions between growth factors and adhesion molecules or cell receptors that mediate adhesion of components.

Many studies using markers for different constituents of EMC were conducted to clarify the role of this structure in the process of carcinogenesis and progression of head and neck tumors.

Collagen represents a family of characteristic proteins with over 20 known types, present in all multicellular animals, being the most abundant components in all extracellular matrixes^2^, ^18^.

Some studies have demonstrated discontinuity of different types of collagen during the process of tissue invasion by neoplastic cells^7^, ^18^.

Studies conducted by Becker et al.^19^ in normal oral mucosa and squamous cell carcinoma highlighted that in normal oral mucosa we can observe a significant amount of collagen III in the subepithelial region and collagen I and II in the deepest portions of connective tissue. In the stroma of oral squamous cell carcinoma, authors noticed that collagen I fibers surrounded the tumor epithelial islands and were distributed throughout the stroma. In laryngeal tumors, Hagedorn et al.^20^ detected defects of peritumor basal membrane owing to loss of significant expressions of collagen IV.

Intensity and quality of collagen I marking presented a varied form in oral squamous cell carcinoma of low and high grade malignancy in the study developed by Martins et al.^18^, in which marking of this type of collagen was more intensive in less aggressive tumors.

Laminin is another protein associated with basal membrane that works as adhesin, binding basal cells of epithelium to collagen IV. It has been directly related with advanced stages of cell differentiation, being a pre-requisite for terminal differentiation and execution of specialized functions, by interacting with integrinins and other components of cell surface as well as controlling cell migration, polarization, proliferation and apoptosis^21^.

According to the reports by Thorup et al.^22^, in poorly differentiated oral squamous cell carcinomas there is marked loss of laminin expression and its integrin receptors, when compared to pre-malignant lesions and inflammatory processes. We evidenced from Tosios et al.^23^ studies a tendency to linear discontinuity of laminin and collagen IV during gradual increase of dysplasia, and in areas of deep tumor invasion more than in central or superficial tumor regions.

Berndt et al.^24^ associated the behavior of oral squamous cell carcinoma with synthesis of laminin, a fact evidenced through focus loss of basal membrane and its deposition in the stroma immediately close to invasive neoplastic cells.

In previous studies, Harada et al.^25^ found a pattern of expression similar to laminin, collagen IV and heparan sulfate in the invasive front of oral carcinomas and metastasis in lymph nodes, leading to the conclusion that cell population in deeper areas of the tumor may be determinant to the invasive and metastatic potential of carcinomas situated in this region.

Kannan et al.^26^ noticed in the oral cavity a gradual increase of frequency of laminin discontinuity and collagen IV from normal, hyperplastic, dysplastic epithelium to malignant one, reporting that progressive affection of basal membrane in malignant transformation may be an indicator of clones of cell population with invasive phenotype, despite the fact that it had not evidenced in the study any correlation between distribution of laminin and collagen IV with grade of malignancy. This trend was also previously noticed in neck epithelial neoplasms and aerodigestive tract dysplasias^23^. The reduced expression of collagen IV was also noticed by Harada et al.^25^ in oral carcinomas.

In percentage terms, Haas et al.^27^ using immunofluorescence technique, detected that the intensity of laminin on the basal membrane of normal oral mucosa was about 99 to 100%, increasing to 107 to 141% in hyperplastic lesions, significantly reducing the front of oral squamous cell carcinomas (35 to 74%).

As to fibronectin, an adhesive glycoprotein of ECM, it has been suggested that its loss during carcinogenesis may allow free migration of neoplastic cells^21^, ^28^, ^29^. Its expression has been related to poor prognosis of head and neck tumors, a fact confirmed by the studies developed by Kannan et al.^18^ in the normal oral mucosa and squamous cell carcinomas, and by Zidar et al.^30^ who detected gradual increase of fibronectin on basal membranes of laryngeal squamous cell carcinomas, detecting also expression which was inversely proportional to density of associated inflammatory infiltrate and level of tumor differentiation.

Intracytoplasmatic immunomarking of fibronectin was observed in 16.7% of tongue squamous cell carcinomas, contrarily to the lips, in Miranda^31^ study, which may, according to the author, be indicative that tumor cells and not only stromal cells may synthesize fibronectin, building up on the basal membrane and peritumor matrix to probably facilitate adherence and posterior migration through tumor stroma.

Tenascin is an adhesive glycoprotein of ECM expressed in epithelial-mesenchymal membrane during embryogenesis and tumorgenesis^17^, ^32^. This protein had many domains of binding, mediating cell-cell adhesion, cell migration, as well as cell adhesion close to matrix through fibronectin binding to proteoglicans^11^.

When this protein is produced by malignant neoplastic cells, there seems to be an increase in proliferation and migration, probably owing to the fact that it has anti-adhesive properties, because it blocks binding of fibronectin to cells. Such property was detected in the studies by Huang et al.^33^ that described a model in which tenascin impaired the adhesive function of fibronectin by biding to FNH13, inhibiting the co-receptor function of sidecan-4 in the signalization of integrins induced by fibronectin.

It has been demonstrated that its expression is increased in many neoplastic lesions^33^, ^34^. The combination of tenascin and other growth factors, in addition to affections to receptors of neoplastic cells may act synergistically in the modulation of cell growth, interfering in tumor progression, invasive behavior and metastatic potential^17^.

In some neoplasm such as breast and astrocytoma, the high expression of tenascin was indicative of proliferative activity and tumor aggressiveness^35^, but no correlation was observed in salivary gland tumors and some basal carcinomas^36^.

The intracytoplasmatic presence of tenascin in the front of invasion of tongue and jugal mucosa squamous cell carcinoma was detected in the studies by Mori et al.^37^. Yoshida et al.^34^ observed significant increase of expression of tenascin in tumor stroma cells of laryngeal carcinoma especially around neoplastic cell nests, leading the authors to the conclusion that tenascin produced in tumor cells could be involved in intraepithelial extension and invasion of connective tissue, through high mitotic activity and migration of cancer cells.

Upon studying hypopharynx and laryngeal carcinomas, Juhász et al.^11^ noticed that in advanced tumors tenascin was not expressed only in tumor-host interface, but also in the blood vessels, both in stroma and tumor parenchyma. In this study, they confirmed that accumulation of tenascin in the blood vessels could be considered as an indicative factor of poor prognosis of hypopharynx and larynx carcinomas.

Miranda^31^ analyzed the correlation of expression pattern of many proteins in ECM, such as laminin, collagen I and IV, fibronectin and tenascin in lower lip and tongue squamous cell carcinoma with varied histology grading, and observed that collagen IV and laminin were absent on the basal membrane of neoplastic cell nests of most studied cases and when present, marking was weak. Fibronectin was immunomarked in all studied cases and tenascin expressed intensively on epithelial basal membrane in most studied cases, also present in peritumor stroma, predominantly with weak intensity in the lip and moderate to strong in the tongue. To collagen I, it was observed focal expression in both groups of carcinoma, with weak intensity of reaction and predominantly fibrillar and disorganized pattern. The author suggested then that tongue squamous cell carcinomas have greater invasive potential and more aggressive behavior when compared to lower lip tumors.

### Degradation of ECM

Degradation of ECM is an essential event in many physiological processes such as during embryonic development, growth and tissue repair. Conversely, its excessive degradation may result in development of many different pathological conditions, among which we can include rheumatoid arthritis, osteoarthritis, and autoimmune diseases^38^. This fact is also directly related to the process of invasion and tumor metastasis, which is the main prognostic factor related to patients with cancer^39^.

Some specific proteases known as metalloproteinase (MMP) or matrix metalloproteinase, form an important family of metal-dependent endopeptidase, inactively secreted with zinc in the active site^5^, ^38^.

There are currently at least 20 types of human MMPs, which are grouped according to the structure and specific substrate in: collagenase (MMP-1, -8 e 13), stromelisin, gelatinase (MMP-2, -9) and plasma membrane-binding metalloproteinases^12^, ^40^.

According to Curran, Murray^5^ and Nabeshima et al.^40^, overexpression of MMPs has been evidenced and related to prognosis in different types of carcinomas, which can be used for the development of protein inhibiting drugs to support anti-cancer therapy.

It is defined in the literature that tumor invasion also suffers direct influence of the microenvironment, in which one of the critical factors is the action of many different proteolytic enzymes that act directly on degradation of ECM proteins, named metalloproteinases (MMPs)^41^, ^42^, ^43^. These enzymes may be produced both by tumor stroma and by their own neoplastic cells, which may also, according to Yves and Derderck^3^, interfere in the process of cell-cell adhesion by degradation of E-caderin.

To Thomas et al.^12^, the increase in production of these enzymes has been associated with invasive phenotype in many different tumors. Such statement was confirmed in the studies by Hong et al.^1^, who studied expression of MMP-2 and -9 in oral squamous cell carcinomas and correlated presence of MM-9 and metastatic potential of carcinoma, in agreement with the studies by Miyajima et al.^42^ Davies et al.^44^ also detected the presence of metalloproteinases in cancer cells of stomach, breast, liver, and pharynx, relating this fact to malignant potential of these cells and to poor clinical course of these neoplasms.

Many studies developed, such as those reported by O-Charoenrat et al.^45^ and O-Charoenrat et al.^46^, demonstrated that expression of MMPs and its inhibitors (TIMPs) is present in head and neck squamous cell carcinomas. In these studies, the authors noticed that MMPs-2, -7, -9 and –11 played an important role in the progression and development of metastases in these tumors.

Upon analyzing 25 non-metastatic oral squamous cell carcinoma and 19 metastatic carcinomas using immunohistochemical and zymography techniques, Hong et al.^1^ compared expression and activity of MMPs-2 e –9 in these groups and detected that MMP-9 would play an even bigger role in the development of metastases than MMP-2. Additionally, the study did not show correlation between histological grading of carcinomas and expression of MMPs, suggesting that histology differentiation of cancer cells does not interfere in expression of MMPs.

Nabeshima et al.^40^ reported that MMP-2 and -4 are capable of cleavage site g^2^ of laminin-5, leading to stimulation of proliferation and migration of neoplastic cells. These authors also reported that metalloproteinases interfere in the cell-cell adhesion mechanism especially over the complex E-caderin/b-catenin, facilitating the invasion process.

The importance of knowing the role of ECM and MMPs proteins in head and neck carcinomas is quite obvious, given that they play a key role in progression and aggressiveness of these tumors.

## CLOSING REMARKS

In view of the studied literature, we observed that ECM and MMPs, owing to their influence on many cell functions during carcinogenesis and because they are directly related with behavior of head and neck carcinoma, deserve special attention when being studied, given that ECM and its degradation represent the key element in understanding the mechanisms involved with tumor invasion. Affections to extracellular matrix through overexpression of tenascin or discontinuity in collagen, fibronectin and laminin structure, in addition to the presence of MMPs, influence the behavior of neoplastic cells, acting over tumor growth, tissue adhesion, invasion and metastases, which represent the main factors indicative of prognosis and survival of patients with these carcinomas.
